# A Review and Database of Snake Venom Proteomes

**DOI:** 10.3390/toxins9090290

**Published:** 2017-09-18

**Authors:** Theo Tasoulis, Geoffrey K. Isbister

**Affiliations:** Clinical Toxicology Research Group, University of Newcastle, Newcastle 2298, Australia; Theodoros.Tasoulis@uon.edu.au

**Keywords:** snakes, venom, proteomics, elapid, viper, toxins

## Abstract

Advances in the last decade combining transcriptomics with established proteomics methods have made possible rapid identification and quantification of protein families in snake venoms. Although over 100 studies have been published, the value of this information is increased when it is collated, allowing rapid assimilation and evaluation of evolutionary trends, geographical variation, and possible medical implications. This review brings together all compositional studies of snake venom proteomes published in the last decade. Compositional studies were identified for 132 snake species: 42 from 360 (12%) Elapidae (elapids), 20 from 101 (20%) Viperinae (true vipers), 65 from 239 (27%) Crotalinae (pit vipers), and five species of non-front-fanged snakes. Approximately 90% of their total venom composition consisted of eight protein families for elapids, 11 protein families for viperines and ten protein families for crotalines. There were four dominant protein families: phospholipase A_2_s (the most common across all front-fanged snakes), metalloproteases, serine proteases and three-finger toxins. There were six secondary protein families: cysteine-rich secretory proteins, l-amino acid oxidases, kunitz peptides, C-type lectins/snaclecs, disintegrins and natriuretic peptides. Elapid venoms contained mostly three-finger toxins and phospholipase A_2_s and viper venoms metalloproteases, phospholipase A_2_s and serine proteases. Although 63 protein families were identified, more than half were present in <5% of snake species studied and always in low abundance. The importance of these minor component proteins remains unknown.

## 1. Introduction

Medically significant venomous snakes are almost entirely front-fanged, and are classified into three families: Atractaspidae (Burrowing Asps, 69 species), Elapidae (Elapids, 360 species), and Viperidae (Vipers, 340 species). This last family is in turn divided into two subfamilies, Viperinae (True Vipers, 101 species), and Crotalinae (Pit Vipers, 239 species) (data taken from www.reptile-database.org). The venom glands of caenophidian (advanced) snakes are homologous [1], and current evidence suggests that the three families of front-fanged snakes evolved from non-front-fanged venomous snakes [2].

Snake venoms are mixtures of different protein families, and each of these families contains many different toxins or toxin isoforms. As snake venom glands are homologous, it would be expected that some toxin families would be ubiquitous across the three front-fanged snake families. This ancestral venom proteome has since diversified among different snake families due to the influence of genetic mutations, genetic drift, and natural selection differentially molding the venom of each species to confer optimal prey specific toxicity.

For decades, a major line of research in snake venom studies has been investigating the structure and function of single toxins. Recent advances in the last decade in transcriptomics technology, combined with well-established proteomics methods such as reverse-phase high performance liquid chromatography (RP-HPLC), and mass spectrometry (MS), has enabled rapid identification of different toxins in snake venoms, as well as the ability to rapidly measure their relative abundance. These technological advances have fortuitously coincided with major improvements in our understanding of snake evolutionary relationships (phylogeny). As the venom proteomes of over 100 snake species have now been published, there is a sufficient number of studies to allow the general themes in snake venom evolution to begin to be understood. There is a need to collate this data for each family/subfamily of snakes for comparative analysis. This review collects all the studies published in the last ten years that provide relatively complete compositional abundances of the toxins in snake venoms. It could thenform the basis of an online database to be continually expanded as the venom profiles of more snake species are added to the body of knowledge.

## 2. Results

**Figure 1:**
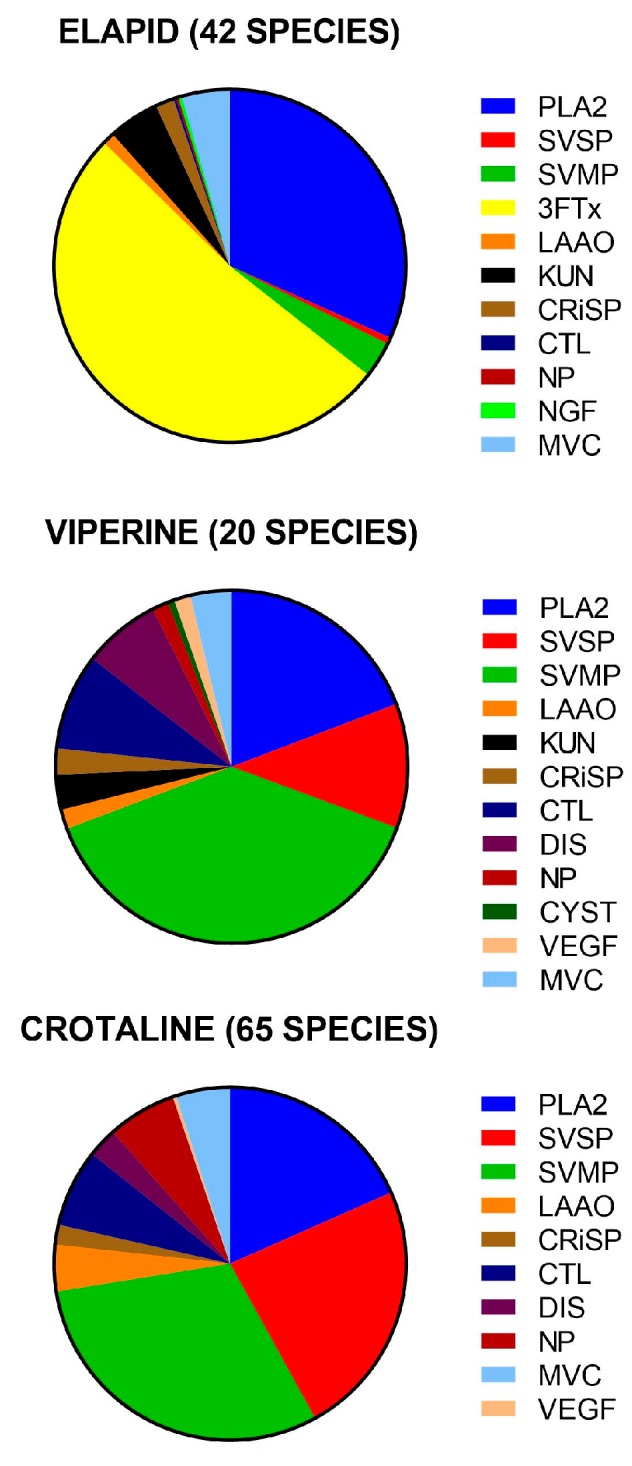
The relative proportions of different protein families for the venoms of: elapids (**upper**); viperines (**middle**); and crotalines (**lower**), averaged from the number of species noted in the brackets. **PLA_2_**, phospholipase A_2_; **SVSP**, snake venom serine protease; **SVMP**, snake venom metalloprotease; **LAAO**, l-amino acid oxidase; **3FTx**, three-finger toxin; **KUN**, kunitz peptide; **CRiSP**, cysteine-rich secretory protein; **CTL**, C-type lectin; **DIS**, disintegrin; **NP**, natriuretic peptide; **NGF**, nerve growth factor; **CYS**, cystatin; **VEGF**, vascular endothelial growth factor; **MVC**, minor venom component.

Compositional venom studies were identified for 132 species of snakes: 42 species from 360 (12%) Elapididae (elapids), 20 species from 101 (20%) Viperinae (true vipers), 65 species from 239 (27 %) Crotalinae (pit vipers), and five species of non-front-fanged snakes (percentage unknown—perhaps <3%). A total of 63 protein families were identified in all of the studies in the venoms of the 130 snake species reviewed. Of the 127 species of front-fanged snakes, their venom contained 59 protein families. For this group, with only a few exceptions, approximately 90% of their total venom composition was made up of eight protein families for elapids (Table 1 and Figure 1), 11 protein families for viperines (Table 2 and Figure 1), and ten protein families for crotalines (Table 3 and Figure 1). Three species (two elapids and one crotaline) had unusual venom compositions (Tables S1 and S2).

The 59 protein families could be classified based on compositional abundance and ubiquity. These categories were: four dominant protein families: phospholipase A2s (PLA_2_), metalloproteases (SVMP), serine proteases (SVSP) and three-finger toxins (3FTx); six secondary protein families: cysteine-rich secretory proteins (CRiSP), l-amino acid oxidases (LAAO), kunitz peptides (KUN), C-type lectins/snaclecs (CTL), disintegrins (DIS) and natriuretic peptides (NP); nine “minor” protein families; and 36 “rare” protein families (Table S3). There was also a further group of four “unique” protein families, which were each restricted to a single genus. 

**Figure 2:**
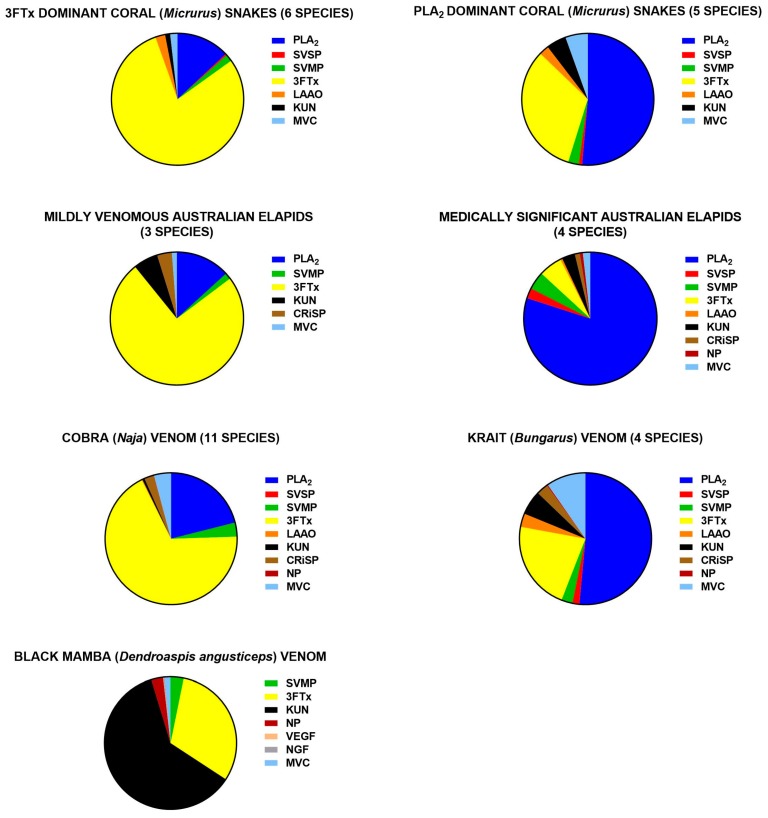
Differences in the venom composition among the family elapidae, averaged from the number of species noted in the brackets. The 3FTx/PLA_2_ dichotomy is shown for New World coral snakes (upper pair), Australian elapids (middle pair) and Afro-Asian cobras and kraits (lower pair). The lowermost pie chart shows the unique venom composition of African black mamba. **PLA_2_**, phospholipase A_2_; **SVSP**, snake venom serine protease; **SVMP**, snake venom metalloprotease; **LAAO**, l-amino acid oxidase; **3FTx**, three-finger toxin; **KUN**, kunitz peptide; **CRiSP**, cysteine-rich secretory protein; **NP**, natriuretic peptide; **VEGF**, vascular endothelial growth factor; **NGF**, nerve growth factor; **MVC**, minor venom component. Mildly venomous Australian species: *Drysdalia coronoides*, *Austrelaps labialis* and *Toxicocalamus longissimus*. Medically significant Australian elapids: *Oxyuranus scutellatus*, *Notechis scutatus*, *Pseudechis papuanus* and *Micropechis ikaheka*.

The major difference between elapid and viper venoms was the presence of 3FTx in elapid venoms and the virtual absence of 3FTx in viper venoms. Elapid venoms were also less diverse in the range or number of protein families, largely consisting of only PLA_2_ and 3FTx, although different groups were dominated by one or the other (Figure 2). Elapid venoms were more variable in the amount of different protein families compared to viper venoms.

Protein families in non-front-fanged snakes are included in Table S4.

## 3. Discussion

A total of 63 protein families were identified in the venoms of the 132 snake species included in this review. As the venom composition of only five species of non-front-fanged snake species were found, we will focus on the 127 species of front-fanged snakes, which contain 59 different protein families. Despite this diversity, with only a few exceptions, more than 90% of elapid and viper venoms were composed of just ten protein families (Figure 1). Based on their compositional importance and ubiquity, these 59 protein families were classified into five groups.

1. Dominant protein families (four families): PLA_2_, SVMP, SVSP and 3FTx.
2. Secondary protein families (six families that were commonly present, but in much smaller amounts than the dominant families): KUN, CRiSP, LAAO, CTL, DIS, and NP.
3. Minor protein families (nine families): acetylcholinesterase, hyaluronidase, 5′ nucleotidase, phosphodiesterase, phospholipase B, nerve growth factor, vascular endothelial growth factor, vespryn/ohanin and snake venom metalloprotease inhibitor.
4. Rare protein families: 36 families listed in Table S3.
5. Unique protein families (four families): defensins, waglerin, maticotoxin and cystatins. These families make up to 38% of the whole venom of a single species, but are classified separately as each is present in only one genus.

Both elapid and viper venoms were dominated by two or three protein families, PLA_2_s and 3FTxs for elapids and SVMPs, PLA_2_s and SVSPs for vipers (Figure 1). These protein families made up on average 83% and 67% of the venom proteome of elapids and vipers, respectively. Viper venoms consisted mainly of PLA_2_, SVMP and SVSP, but the variability in the amounts of different protein families between different groups of vipers was less than for elapids.

There was then a secondary group of six protein families, which made up 11% and 22% of the venom proteome of elapids and vipers, respectively. The remainder of the venoms consisted of minor abundance protein families belonging to nine minor protein families and 36 rare protein families, which were only present in a few species and in small amounts (nearly always less than 2%) (Table S3). It is unknown if these protein families are vestigial relics of snake evolutionary history (redundant, due to acquired prey immunity), or are recent genetic mutations. 

There were four unique protein families, each only present in one genus but often making up the dominant fraction in the venom: Defensins, *Crotalus*; Waglerin, *Tropidolaemus*; Maticotoxin, *Calliophis*; and Cystatins, *Bitis.* Cystatins placement in this category was somewhat arbitrary, as it only comprised 2–10% of the venom, but was present in four of the five *Bitis* species studied and in no other snake species apart from the extremely aberrant *Calliophis*.

### 3.1. Dominant Protein Families

**Phospholipase A_2_s**. This was the most widespread protein family in elapids and vipers. However, the type of PLA_2_ differed between families with pancreatic type I in elapids and synovial-type II in vipers [99]. Maximum recorded amounts were 90% for *Pseudechis papuanus* (elapid), 51% for *Agkistrodon contortrix* (crotaline) and 65% for *Vipera nikolskii* (viperine).

**Three-finger toxins**. These toxins were only present in elapids (except for one Crotaline *Atropoides nummifer* <0.1%). In elapids, they made up to 95% of the total venom (*Micrurus tschudii*) and were present in 98% of all species. 3FTxs have been only recorded in small quantities in several viper species in other studies; *Lachesis muta* [100], *Sistrurus catenatus* [101], *Protobothrops* [54] and *Daboia russelii* [102].

**Metalloproteases**. This was the major protein family in viper venoms, present in all of the viper species included. The maximum amounts present were 72% for viperines (*Echis ocellatus*), and 85% for crotalines (*Bothrops atrox*). They were of lesser importance in elapids. Although they were present in 88% of species, they made up a much smaller proportion of the venom; maximum amounts recorded were 19% for *Calliophis bivirgata* and 12% for *Ophiophagus hannah*.

**Serine proteases**. These were the least quantitatively important of the dominant protein group. They were present in almost all vipers with the maximum amounts being 31% (*Vipera berus*) and 93% (*Ovophis okinavensis*). They were only present in 29% of elapids with the maximum amount being 6% for *Notechis scutatus*. However, they may potentially make up to 15% in some other Australian elapids, such as *Pseudonaja textilis* (Tasoulis et al. unpublished), because there are few proteomic studies of Australian elapids and it is well known that SVSPs are of major importance in Australian snakes as procoagulants [103,104].

### 3.2. Secondary Protein Families

**Kunitz peptides**. This family was the major venom component in black mambas (61%). This protein family was entirely absent from crotalines and, in viperines, was common in only three genera: *Bitis, Macrovipera* and *Daboia*. As crotalines are a derived viperine, this suggests that their absence in crotalines is the result of a reversal or secondary loss. Amongst other elapids, they may make up to 13% of the total venom. 

**l-amino acid oxidases**. These enzymes were most common in crotalines in both the number of species that contained them (91% of all species) and in proportion of a single species venom with a maximum of 20% in *Rhinocerophis cotiara*. They were of relatively equal importance in elapids and viperines, always present in more than half the species and in amounts up to 6%.

**Cysteine-rich secretory proteins**. This group was widespread across all families, but more common in vipers (88% of species) than elapids (56% of species). The maximum amounts recorded in individual snake venoms were 10% for elapids and 16% for vipers. 

**C-type lectins/snaclecs**. These were only a minor component of elapid venoms (maximum amount 2%), but were present in 100% of viperine venoms with a maximum amount 22% in *Daboia russelii* and 88% of crotalines with a maximum amount of 31% in *Bothrops insularis*. 

**Disintegrins**. This protein family was entirely absent in elapids and was of relatively equal importance in viperines (88% of species) and crotalines (68% of species). The maximum amounts were 18% and 17%, respectively, for viperines and crotalines.

**Natriuretic peptides**. This protein family was far more important in vipers than elapids. They were only recorded in 20% of elapids with a maximum amount of 3% in *Dendroaspis polylepis*. They were present in 35% of viperines with a maximum amount of 11% in *Vipera berus* and in 60% of crotalines with a maximum amount of 37% in *Bothriechis nigroviridis*.

### 3.3. Major Inter-Family Differences

A major difference between elapids and vipers was the virtual absence of 3FTxs from viper venoms, while being one of the two dominant protein families in elapid venoms. As noted by Aird et al. [54], while 3FTxs have been recorded from several viper venoms, it has been via transcriptomics studies, not proteomic approaches. Vespryns/ohanins were only recorded in elapid venoms, with only three exceptions, none exceeding 0.5% (*Agkitrodon bilineatus, Bothropoides pauloensis* and *Crotalus viridus*—all crotalines). Conversely, DISs were absent in elapids while occurring in almost all viper venoms (averaging 2–6%). Another protein family conforming to this trend was CTLs, which again were a common component in viper venoms (averaging 6–8%), but were a minor component in elapid venoms, only present in about a third of the species, and in amounts not more than 2%. A similar trend was also apparent for NPs.

#### 3.3.1. Elapids

Remarkably, for about 90% of the elapids, greater than 75% of their total venom composition was made up of just two protein families: PLA2s and 3FTxs. Lomonte et al. [32] drew attention to the divergence in venom phenotypes exhibited by New World coral snakes (*Micrurus*). There is a sharp dichotomy among species in the relative proportions of PLA_2_s and 3FTxs in their venoms. Our study shows that there is also a dichotomy in the proportion of PLA_2_s and 3FTxs in elapids on the Australian continent (Figure 2). The medically important Australo-Papuan elapids (*Elapidae: Hydrophiinae*) are dominated by PLA_2_s and the smaller and less important elapids contain mainly 3FTxs, similar to the Afro-Asian cobra venoms which are also mainly 3FTxs. Lomonte et al. [32] suggested that based on the known phylogeny, 3FTxs could be the ancestral state in coral snakes, with an evolutionary trend towards a greater preponderance of PLA_2_. Interestingly, when mapped onto the currently accepted phylogenies, all the most basal species in every clade of Australo-Papuan elapids have venom that is predominately composed of 3FTxs, while the derived species possess venom dominated by PLA_2_s. This suggests that this trend has happened repeatedly in the Australia–New Guinea region: New Guinea terrestrial elapids [105], Australian terrestrial elapids [106,107] and sea snakes [108,109]. However, this is based on the proteomics of only a few species and requires further investigation.

Kraits (*Bungarus*) are also dominated by PLA_2_s, making up almost half the venoms with less 3FTxs, similar to the medically important Australian elapids. However, they contain larger amounts of the secondary protein families—KUNs, LAAOs and CRiSPs—compared to other elapid groups.

The venoms of most cobra (*Naja*), species are dominated by 3FTxs, with less PLA_2_s. Interestingly, there is a similar dichotomy between cobras and kraits in proportions of PLA_2_s and 3FTxs, to coral snakes and Australo-Papuan elapids (Figure 2). Cobras also lacked many of the secondary protein families, except for CRiSPs, which are present in relatively large amounts in two species (both non-spitting species), *N. haje* (10%) and *N. melanoleuca* (7.6%). The venoms of the African spitting cobras were the most simplistic of all cobra species in the number of different protein families making up their venom.

Apart from *Calliophis*, *Dendroaspis* (Mambas), had the most unique venoms of the elapids, having no PLA2s, but instead containing specialized KUNs (dendrotoxins—Kv1 channel blockers) and 3FTxs modified into both acetylcholinesterase inhibitors (fasciculins) and l-type calcium channel blockers (calciseptines). Additionally, the KUNs are present in *Dendroaspis* in far greater amounts than in any other elapid species. 

The most aberrant venom displayed by any elapid species was the Malayan blue coral snake *Calliophis bivirgata flaviceps,* which possessed the highest amounts of vespryn/ohanin of any elapid species (14%) and unusually high amounts of SVMPs for an elapid (19%). Even more unusual, almost a quarter of its venom consisted of a unique protein family, maticotoxin A, a cytotoxin. The highly aberrant venoms of these genera could indicate an ancient phylogenetic divergence.

#### 3.3.2. Vipers

Viperine and crotaline venoms were quite similar, being composed mainly of three dominant protein families: PLA_2_s, SVMPs and SVSPs (Figure 1). The major difference between the two subfamilies was that KUNs were present in viperines and absent in crotalines. Additionally, crotalines possessed glutaminyl cyclases which were absent from viperine venoms. Some protein families were restricted to a single genus, such as defensins (crotamine), which were only found in the crotaline genus *Crotalus* (rattlesnakes), while cystatins were only found in the viperine genus *Bitis*. Both of these were present in significant amounts. The results confirmed the longstanding paradigm that viperid venoms consist of predominately hemotoxins, hemorrhagins, myotoxins and cytotoxins. 

**Figure 3:**
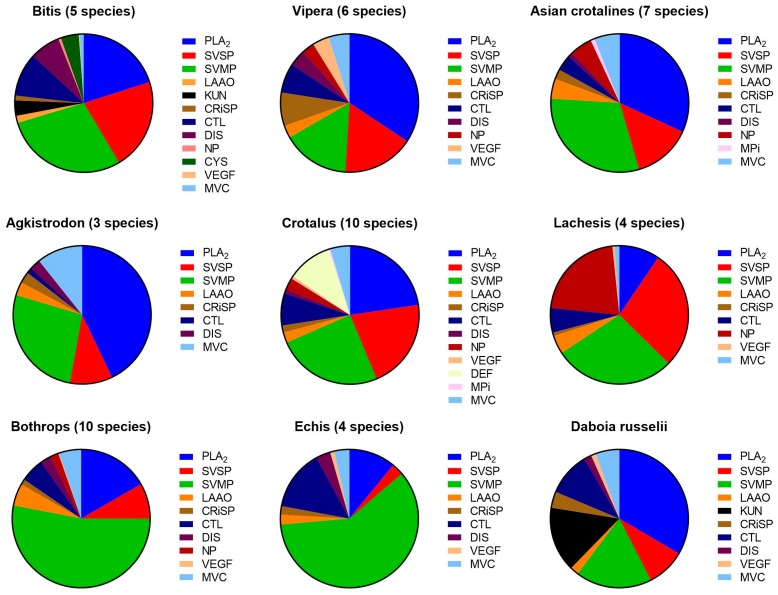
Differences in venom composition in different genera of viperids, showing the less extreme variation in individual protein families compared to elapids. The majority of the venoms are made up of SVMP, PLA_2_ and SVSP. Differences include the presence of KUNs in viperines and the greater importance of NPs in some crotalines. Abbreviations: **SVMP**, snake venom metalloprotease; **PLA_2_**, phospholipase A_2_; **SVSP**, snake venom serine protease; **LAAO**, l-amino acid oxidase; **CRiSP**, cysteine rich secretory protein; **CTL**, C-type lectin/snaclec; **DIS**, disintegrin; **NP**, natriuretic peptide; **VEGF**, vascular endothelial growth factor; **MVC**, minor venom components_._

Few obvious trends were discernible in crotaline venoms at a protein family level, with less variability across the family (Figure 3). *Lachesis* has generally higher amounts of NPs than most crotalines, in addition to having relatively large amounts of SVSPs. The meso-american genera *Atropoides*/*Cerrophidion* and *Porthidium*, all possessed noticeably large amounts of DIS, CRiSPs and CTLs. Asian crotalines could not be separated from American crotalines based on venom protein families, suggesting an interesting wide geographical similarity (Figure 3).

The most aberrant venom of any viperid was *Tropidolaemus*. In addition to having 38% of its venom being represented by a unique protein family waglerin, the three dominant protein families in other viperid venoms made up only 15% of its venom proteome.

### 3.4. Medical Implications

PLA_2_s are present in 95% of the two most medically important snake families (elapids and vipers), excepting the limitation that there are some important groups of snakes that have not been investigated. Having a common protein family so widely spread has useful implications, such as making it possible to develop an assay that tests for the presence of snake venom in human body fluids. This has been shown for a limited number of snakes, in which measurement of phospholipase activity in patient samples identified patients with viper and elapid envenomation [110].

Demonstrating that there is a limited number of protein families in snake venoms, which is even more limited for major snake families or sub-families, supports efforts to develop universal anti-venoms [111].This explains the cross-neutralization of venoms by different anti-venoms, such as Asian and Australian anti-venoms cross-neutralizing neurotoxicity [112], and cross-neutralization of pit-viper venoms [113].

### 3.5. Evolutionary Biology

It has been known for some time that snake venoms are homologous and restricted to a small number of clearly successful protein families [114,115]. These molecular scaffolds have undergone considerable evolutionary “tinkering” to maximize their lethality to prey. Due to its finer inter- and intra-genus level resolution, this review represents a new and complementary dataset which can supplement future research in evolutionary biology. Although the extreme potency of some snake venoms clearly argues for powerful positive directional selection, it is also highly likely that in many adaptive radiations of snakes venom variation is not the primary driver of speciation. New ecological opportunities are also important e.g., the unidirectional late Oligocene or early Miocene invasion of the Americas by crotalines and elapids [116], and climatic oscillations can act as engines of speciation (“species pump”) [117,118,119]. Venom is simply one of many competing traits being selected for [120], and is often of neutral selective value [121,122]. Therefore, toxin evolution cannot be considered in isolation to other species traits.

**Figure 4:**
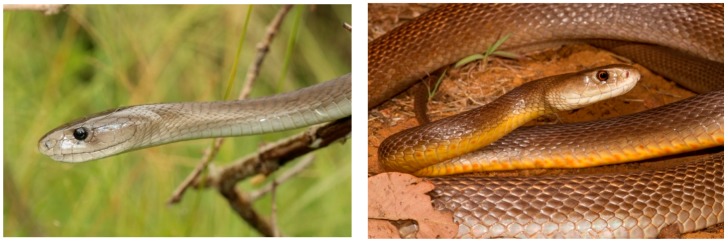
African black mamba *Dendroaspis polylepis* (**left**) and Australian coastal taipan *Oxyuranus scutellatus* (**right**): These elapids represent evolutionary parallels on different continents in terms of their morphology, ecology and biology, but the pharmacological effects caused by their venoms are the result of different protein families. Some of these protein families have convergently evolved to cause potent neurotoxicity. Photo credits: Nick Evans (black mamba), and Brendan Schembri (coastal taipan).

An additional consideration is that different protein families can be equally effective in immobilizing the same prey type. A classic example of this is comparing the African elapid, black mamba *Dendroaspis polylepis* and the Australian elapid, coastal taipan *Oxyuranus scutellatus*. As noted by Shine [123] these two species represent a remarkable instance of parallel evolution resembling each other in body size, general morphology (Figure 4), color, venom toxicity, fang length, “snap and release” bite, clutch size, hatchling size, rapid growth in juveniles, males growing larger than females and feeding primarily on mammals. Despite having the same diet, they have a completely different composition of protein families in their venom. Black mamba contains mostly KUNs and 3FTxs, while coastal taipans have PLA_2_s and SVSPs. About 25% of coastal taipans completely lack 3FTxs in their venom or they are present in very small amounts (Tasoulis et al. unpublished). To further compound the difference, taipan toxins are mainly enzymatic while mamba venoms are almost entirely non-enzymatic. However, some of these protein families have converged to target the central nervous system, although in different ways [124,125,126,127], while others have evolved to target different physiological systems, such as the procoagulant (prothrombin activator), in coastal taipan venom. Black mamba venom is devoid of coagulopathic enzymes [18].

Previous evolutionary studies on snake venom have investigated phylogeny-based comparisons [128], co-evolution of venom and prey [94,129,130,131,132,133], prey resistance to venom [134,135], gene loss and duplication [136,137,138,139], exon exchange [3,101,140] and venom ontogeny [141,142,143,144,145,146,147]. This database allows us to study snake venom evolution through deep time. It can be used in conjunction with studies aimed at unraveling the historical biogeography of particular lineages of venomous snakes that, due to the known geological and climatic history of their in situ evolution, represent model case studies of evolution. These studies can examine venom evolution at different hierarchical levels, inter and intra-generic, as well as intra-specific.

Examples of this are the recent studies done on Meso-American pit vipers *Atropoides, Bothriechis,* and *Cerrophidion* [148,149] that are providing strong support for an underlying biogeographic model which gives a robust framework for estimating temporal rates of change and divergence times. Ideally, the venoms of these snake genera would be studied in even finer resolution, not just at a protein family level, but a complete venom profile of all toxins and toxin isoforms. This data can then be compared with probable divergence times to show which toxins are undergoing accelerated evolution. Due to its enormous diversity, and lability, venom could prove to be a phenotypic trait par excellence for studying evolutionary biology.

Although the relative abundance of protein families can be rapidly altered by genetic drift, (e.g., founder effect), some conserved toxins may act as reliable biomarkers for tracing evolutionary history. The venom compositions of the species in the viperine genus *Bitis* (Table 2) show a perfect congruence with the phylogenetic relationships of this genus proposed by Wittenberg et al. 2015 [150]. There is also evidence that snakes with unique venom represent species of ancient phylogenetic divergence (e.g., *Calliophis* and *Dendroaspis* [151,152] and *Tropidolaemus wagleri* [153].

Dowell et al. [136], have shown how rattlesnake venoms can become simplified due to gene deletions resulting in the loss of toxins. Our data analysis also reveals numerous examples where protein groups appear to have been lost in individual species and entire genera. For example LAAOs and CRiSPs are present in all *Bitis* species examined except *B. arietans.* NPs and vascular endothelial growth factors are absent in all *Bitis* species examined except the sister species *B. gabonica*/*rhinoceros*. Given the homologous nature of these protein families and their ubiquity, these patterns are difficult to explain other than being the result of character reversals. 

Finally, on broader scale, trends in snake venom evolution can be compared with venom evolution in other groups of venomous organisms.

## 4. Methods

An online search was conducted using MEDLINE (PubMed platform) and Google Scholar from May 2006 to September 2017 using the keywords “snake venom proteomics”, and “snake venomics”. In addition, because of their strong emphasis on publishing articles on snake venom, the contents of the following journals were searched for articles on snake venom proteomics published between May 2006 and September 2017: Journal of Proteome Research, Journal of Proteomics, Toxicon, and Toxins. In addition, the journal BMC Genomics was searched using the keywords “snake venom proteomics”. The reference lists for any studies found were then searched for additional studies. The search was restricted to English language publications. Only studies that showed the compositional abundance of each protein family were included. If multiple studies had been conducted on a single snake species, the most recent one (which usually had the finest resolution) was included. To eliminate possible differences caused by ontogenetic venom variation, only data for adult snakes was considered. In cases in which venom from a particular snake species had marked geographical variability, entries for each geographical region were incorporated. For transcriptomics studies, data was usually presented as both relative expression of toxin-encoding genes and genes detected in the venom gland by proteomics. In these instances, the proteomic data was used. Many studies did not use transcriptomics, but instead a combination of reverse-phase high performance chromatography and electrophoresis followed by trypsin digestion and mass spectrometry. Researchers often did not state the number of individuals used in the study; presumably, many studies were based on the venom of a single snake. Due to space limitations, as well as ease of assimilating the data, the tables presented in the Results Section include only the protein families that make up the majority of the total venom proteome. For convenience, snake species with anomalous protein families in their venom were placed in separate tables for each snake family/subfamily. The remaining minor abundance protein families were included as a separate list.

## Figures and Tables

**Table 1:** The 42 elapids included in the study (excluding two aberrant species), with the proportion of each of the eight major protein families in venom (expressed as percent of total venom), which make up 80–100% of their venom proteome.

SPECIES	PLA_2_	SVSP	SVMP	LAAO	3FT	KUN	CRiSP	NP	%WV	3FT + PLA_2_	REF
*Austrelaps labialis*	33		3		45	9	8		98	78	[3]
*Drysdalia coronoides*					86.4	9.2	2.8		98.4	86.4	[4]
*Micropechis ikaheka*	80	<0.1	7.6	0.4	9.2	0.7	1.8		99.8	89.2	[5]
*Notechis scutatus*	74.5	5.9			5.6	6.9	0.3	2	93.2	80.1	[6]
*Oxyuranus scutellatus*	68–80	<5	5–9		0–9	<10	<1	1	>90	68–89	[7]
*Pseudechis papuanus*	90.2		2.8	1.6	3.1		2.3		100	93.3	[8]
*Toxicocalamus longissimus*	6.5		1.4		92.1				100	98.6	[9]
*Aipysurus laevis*	71.2				25.3		2.5		99	96.5	[10]
*Hydrophis cyanocinctus*	18.9				81.1				100	100	[9]
*H. platurus*	32.9		0.9		49.9		9.1		92.8	82.8	[11]
*H. schistosus*	27.5		0.5	0.2	70.5		1.3		100	98	[12]
*Laticauda colubrina*	33.3				66.1		0.05		99.45	99.4	[13]
*Bungarus caeruleus (Sri Lanka)*	64.5		1.3		19	4.4	5.5		94.7	83.5	[14]
*B. candidus* Malaya	25.2	3.9	4.9	5.8	30.1	12.6	3.9	1	86.4	55.6	[15]
*B. fasciatus* Vietnam	66.8		3.5	7	1.3	1.8	0.4		80.8	68.1	[16]
*B.fasciatus* Malaya	44.2	5.8	4.7	5.8	17.4	9.3	1.2		88.4	61.6	[15]
*Dendroaspis angusticeps*			6.7		69.2	16.3	2		94.2	69.2	[17]
*D. polylepis*			3.2		31	61.1		2.9	95.3	31	[18]
*Naja haje*	4		9	1	60	1.9	10		85.9	64	[19]
*N. melanoleuca*	12.9		9.7		57.1	3.8	7.6		91.1	70	[20]
*N. katiensis*	29		3.3		67.1		0.2		99.6	96.1	[21]
*N. mossambica*	27.1		2.6		69.3				99	96.4	[21]
*N. nigricollis*	21.9		2.4		73.2		0.2		97.7	95.1	[21]
*N. nubiae*	26.4		2.6		70.9				99.9	97.3	[21]
*N. pallida*	30.1		1.6		67.7				99.4	97.8	[21]
*N. atra* China	12.2		1.6		84.3		1.8		99.9	96.5	[22]
*Naja atra* Taiwan	14–17		2–2.6	0.2	76–80		2.2–2.4		>93	90–97	[23]
*N. kaouthia* China	26.9		1.1		56.6		5.4		90	83.5	[24]
*Naja kaouthia* Malaya	23.5		3.3	1.1	63.7	0.5	4.3		96.4	87.2	[25]
*Naja kaouthia* Thailand	12.2		2.6	1	78.3		2.3	0.2	96.4	90.5	[25]
*Naja kaouthia* Vietnam	17.4		1.6	0.5	76.4		0.8		96.7	93.8	[25]
*N. naja* Eastern India	11.4	0.3	1	0.8	63.8	0.4	2.1	2	79.8	75.2	[26]
*Naja naja* North-west India	21.4		0.9		74		2.5		98.8	95.4	[27]
*Naja naja* Sri Lanka	14		0.9		80.5		3.7		99.1	94.5	[27]
*N. sputatrix*	31.2	0.4	1.3	0.1	64.2	0.2			97	95.4	[28]
*Ophiophagus hannah*	2.8		11.9	0.5	64.5	3.3	6.5	0.2	89.5	67.3	[29]
*Micrurus alleni*	10.9		1.2	3	77.3				92.4	88.2	[30]
*M. altirostris*	13.7		0.9	1.2	79.5	2.1	0.1		97.5	93.2	[31]
*M. clarki*	36.5	1	1.6	3.8	48.2	0.9			92	84.7	[32]
*M. corallinus*	11.9	0.8	2.9	2.3	81.7				99.6	93.6	[31]
*M. dumerelii*	52	1.9	1.8	3.1	28.1	9			95.9	80.1	[33]
*M. fulvius*	64.9		2.9		25.1	2.2			95.1	90	[34]
*M. mipartitus*	29	1.3	1.6	4	61.1	1.9			98.9	90.1	[35]
*M. mosquitensis*	55.6	0.5	2.6	2.8	22.5	9.8			93.8	78.1	[30]
*M. multifasciatus*	8.2		3.6	3.2	83	1.9			99.9	91.2	[35]
*M. nigrocinctus*	48	0.7	4.3	2.3	38				93.3	86	[36]
*M. tschudii*	4.1			0.7	95.2	1.6			100	99.3	[37]

Abbreviations: **PLA_2_**, phospholipase A_2_; **SVSP**, snake venom serine protease; **SVMP**, snake venom metalloprotease; **LAAO**, l-amino acid oxidase; **3FT**, three-finger toxin; **KUN**, Kunitz peptides; **CRiSP**, Cysteine-Rich Secretory Protein; **%WV**, percentage of venom; **3FT + PLA_2_**, percentage of whole venom made up of these two protein families.

**Table 2:** The 20 viperines (true vipers) included in the study with the proportion of the 11 major protein families in each venom (expressed as percent of total venom), which make up 90–100% of their venom proteome (except *, venom proteome incompletely characterized).

SPECIES	PLA_2_	SVSP	SVMP	LAAO	CRiSP	CTL/SNACLEC	DIS	NP	KUN	VEGF	CYS	%WV	REF
*Bitis arietans*	4.3	19.5	38.5			13.2	17.8		4.2		1.7	99.2	[38]
*B.caudalis*	59.8	15.1	11.5	1.7	1.2	4.9	2.3		3.2			99.7	[38]
*B.gabonica*	11.4	26.4	22.9	1.3	2	14.3	3.4	2.8	3	1	9.8	98.3	[38]
*B.nasicornis*	20.1	21.9	40.9	3.2	1.3	4.2	3.5				4.2	99.3	[38]
*B.rhinoceros*	4.8	23.9	30.8	2.2	1.2	14.1	8.5	0.3	7.5		5.3	98.6	[38]
*Cerastes cerastes (Morocco)*	19.1	6.9	63.1		0.7	1.7	8.5					100	[39]
*C. cerastes (Tunisia)*	16.6	13.2	55.9	6.2		3.2	4.9					100	[39]
*Daboia russelii (Pakistan)*	32.8	3.2	21.8	0.6	2.6	6.4	0.4		28.4	1.5		97.7	[40]
*D. russelii (West India)*	32.5	8	24.8	0.3	6.8	1.8	4.9		12.5	1.8		93.4	[41]
*D. russelii (Sri Lanka)*	35	16	6.9	5.2	2	22.4			4.6			92.1	[42]
*Echis carinatus sochureki*	7.97	4.58	56.57	1.19	1.99	16.53	7.7			0.4		97	[43]
*E. coloratus*	5.7	3.58	61.41	3.91	5.69	9.45	5.8			0.32		96	[43]
*E. ocellatus*	8.5	1.71	72.43	1.36	0.34	6.46				2.72		93.5	[43]
*E. pyramidium leakeyi*	21.57	1.42	48.94	2.83		24.26				0.28		99.3	[43]
*Macrovipera lebetina (Tunisia)*	5	5.5	63.1			3.2	15.1		3.1	3.3		98.3	[44]
*M. l. obtusa*	14.6	14.9	32.1	1.7	2.6	14.8	11.3	5.3				97.3	[45]
*M. mauritanica*	5.5	8.3	45.4			8.1	13.8	4.5	2.5	4.9		93	[44]
*Vipera anatolica*	8.1	1.6	41.5		15.9	1.1	2		0.3			70.5 *	[46]
*V. berus*	10	31	19	2	8	2	1	11				84 *	[47]
*V. kaznakovi*	41	11	16	4	10	12	0.53			4		94.5	[48]
*V. nikolskii*	65	19	0.66	0.08	0.66	4				8		97.4	[48]
*V. orlovi*	24	24	15	5	12	11	0.56		0.15	4		91.7	[48]
*V. raddei*	23.8	8.4	31.6	0.2	7.4	9.6	9.7	6	0.9	2.4		100	[45]
*V. renardii*	44	8	12	4	8	3	13		0.8	3		95.8	[48]

Abbreviations: **PLA_2_**, phospholipase A_2_; **SVSP**, snake venom serine protease; **SVMP**, snake venom metalloprotease; **LAAO**, l-amino acid oxidase; **CRiSP**, Cysteine-Rich Secretory Protein; **CTL/SNACLEC**, C-type lectins and C-type lectin like; **DIS**, disintegrin; **NP**, natriuretic peptides including vasoactive peptides; bradykinin potentiating and inhibitory peptides; **KUN**, kunitz peptides; **VEGF**, vascular endothelial growth factor; **CYS**, cystatin; **%WV**, percentage of whole venom.

**Table 3:** The 65 crotalines (pit vipers) included in the study (excluding one aberrant species), with the proportion of the ten major protein families in each venom (expressed as percent of total venom), which make up 80–100% of their venom proteome. Taxonomy follows Fenwick et al. 2009 [49].

SPECIES	PLA_2_	SVSP	SVMP	LAAO	CRiSP	CTL/SNACLEC	DIS	NP	DEF	MPi	%WV	REF
*Calloselasma rhodostoma*	4.4	14.9	41.2	7	2.5	26.3					96.3	[50]
*Cryptelytrops purpureomaculatus*	8	12	35	10	6	19		2			92	[51]
*Gloydius brevicaudus*	25	3.7	64.4	0.9	1.1	0.2	4.6				99.9	[52]
*G. intermedius*	9.9	36.2	2.6	13.1	6.2	0.8		25.3			94.1	[53]
*Ovophis okinavensis*	0.65	93.1	4.2	0.62		0.47					99	[54]
*Protobothrops elegans*	77.1	10.4	8	0.5	0.1	0.2					96.3	[55]
*Protobothrops flavoviridis*	55.5	11.8	17.3	3.1	2	0.9		2.6			93.2	[56]
*P.mucrosquamatus*	22.5	10.4	43	2	0.8	3.9	0.8	3.6			87	[57]
*Viridovipera stejnegeri*	24.5	11	43.1	3.3	6	1.5	2.2	1.2			92.8	[57]
*Agkistrodon bilineatus (3 subsp)*	34.3–42	7.6–16.9	24.5–30.8	2.6–4.9	0–5.6	0.4–1.4	2.2–3.1	4.6–8.7			76.7+	[58]
*A. c. contortrix*	50.7	5.85	25	4	2	0.8					88.35	[59]
*A. piscivorus (3 subsp)*	33.6–46	10.1–13.9	21–33.1	0.8–4.5	2–3.5	0.8–3.2	2.2–4.9	5.7–5.9			76.2	[58]
*Atropoides nummifer*	36.5	22	18.2	9.1	1.9	1.3	2.5	8.6			100	[60]
*A. picadoi*	9.5	13.5	66.4	2.2	4.8	1.8	<0.1	1.8			100	[60]
*Bothriechis aurifer*		7.3	35.1	9.5	10.7	16.4	1.4	13.4		3.2	97	[61]
*B. bicolor*	35.2	19.1	8.5	10.8	1	4.4	7.6	3.6		4.6	94.8	[61]
*B. marchi*	14.3	10.1	34.2	1.1	2.8	4.2	6.5	10.6		8.5	83.8	[61]
*B. lateralis*	8.7	11.3	55.1	6.1	6.5			11.1			98.8	[61]
*B. nigroviridis*	38.3	18.4		0.5	2.1			37			96.3	[62]
*B. schlegelii*	43.8	5.8	17.7	8.9	2.1			13.4			91.7	[61]
*B. supraciliaris*	13.4	15.2	6.8	5.9	4.3		1.6	21.9			69.1	[63]
*B. thalassinus*		12.1	39.6	4.3	5.1	11.5	2	10.6		9.9	95.1	[61]
*Bothrocophias campbelli*	43.1	21.3	15.8	5.7	0.9	6.4	0.3	3.9			97.4	[64]
*B. colombiensis*	44.3	<1	42.1	5.7	0.1		5.6	0.8			99.5	[65]
*Bothropoides diporus*	24.1	7.2	34.2	7.4		2.9	1.4	15.9		2.6	95.7	[66]
*B. erythromelaus (5 populations)*	10.1–15.1	4–9.7	32.5–59.9		0.4	8.4–21.6	3.4–8.9	9.3–14.5			68+	[67]
*B. insularis*	10	12.5	30	1.3	1.3	31.3		11.3			97.7	[68]
*B. jaracara (south-east)*	3.7	13.7	35.6	7.2	2.4	9.6	7	16.4			95.6	[69]
*B. jaracara (south)*	20.2	28.6	10.3	8	2.6	9.4	0.2	22.6			100	[69]
*B. neuwiedi*	8.4	8.8	49.9	16.7	2	8.6					94.4	[70]
*B. pauloensis*	31.9	10.5	38.1	2.8	2.2	0.6	1.3	12.4			99.8	[71]
*Bothrops asper (Caribbean coast)*	28.8	18.2	41	9.2	0.1	0.5	2.1				99.9	[72]
*B. asper (Pacific coast)*	45.5	4.4	44	4.6	0.1	0.5	1.4				100	[72]
*B. atrox (Western Para Brazil)*	5.7–7.5	9.7–14.1	46.5–54	8.7–9.4	3.7–4.3	10.2–13.1					84.5+	[73]
*B. atrox (Colombia)*	24.1	10.9	48.5	4.7	2.6	7.1	1.7	0.3			99.9	[74]
*B. atrox (Venezuela)*	7.7–8.5	2.3	85	1.2–1.5	2.8–3.8						99+	[75]
*B. atrox (Peru)*	11	11.1	58.2	10.5	2.4	3.6	3.2				100	[76]
*B. ayerbi*	0.7	9.3	53.7	3.3	1.1	10.1	2.3	8.3			88.8	[77]
*B. barnetti*	6.4	6.7	74.1	0.8	3.1	3.3	5.5				99.9	[76]
*B. caribbaeus*	12.8	4.7	68.6	8.4	2.6		1.7				98.8	[78]
*B. jararacussu*	25.7	12.3	26.2	15	2.2	9.7					91.1	[70]
*B. lanceolatus*	8.6	14.4	74.2	2.8		<0.1					100	[78]
*B. pictus*	14.1	7.7	68			1.1	8.9				99.8	[76]
*B. pirajai*	40.2	7.1	20.7	5.2		9.2	1.4	5.6			89.4	[79]
*B. punctatus*	9.3	5.4	41.5	3.1	1.2	16.7	3.8	10.7			91.7	[80]
*Cerrophidion godmani*	23.4	19.1	32.8	5	4.2	0.5	7.5	5.7			98.2	[81]
*C. sasai*	23.4	19.1	32.8	5	4.2	0.5	7.5	5.7			98.2	[82]
*Crotalus adamanteus*	7.8	20	24.4	5.3	1.3	22.2			16.8		97.8	[83]
*C. atrox*	7.3	19.8	49.7	8	4.3	3.4	6.2	3			100	[84]
*C. basiliscus*	14	11	68					2		4	99	[85]
*C. culminatus*	8.3	10.1	35.5	2.7	1.9	13		1.6	24.4		97.5	[86]
*C. durissus cascavella*	90.9	1.2	<0.1	<0.1	0.9	<0.1	0.2				93.4	[87]
*C. d. collilineatus*	72	1.9	0.4	0.5	1.8	<0.1	0.5		20.8		98	[88]
*C. d. terrificus*	48.5	25.3	3.9								77.7	[89]
*C. horridus*	22.8	58.2	0.1	1.1	0.8	0.22			0.2		82.3	[90]
*C. simus simus*	22.4	30.4	27.4	5.7	1	0.6	1.5	6.5			95.5	[91]
*C. tigris*		26.8	66.2		1.9		0.2				95.1	[92]
*C. tzabacan*	11.1	5.4	18.5	0.5		35.2		4.2	23.5		98.4	[86]
*C. viridis*	7.7–10.2	26.8	10.9–11.4	1.9–2.5	2.1–3.9	1.8–3.3	0.1	6.5–8.2	35.6–38	0.1	93.5+	[93]
*Sistrurus catenatus (3 subsp.)*	31.3-31.9	18.2-24.4	40.6-48.6	1.6-4.2	0.8-10.7		0.9-4.2				93.4+	[94]
*S. miliarius*	32.5	17.1	36.1	2.1	2.9		7.7				98.4	[94]
*Lachesis acrochorda*	2.3	35.1	23.2	9.6	0.9	6.9		21.5			99.5	[95]
*L. melanocephala*	13.4	21	18.9	3.6		7.5		30.2			94.6	[94]
*L. muta muta*	8.7	31.2	31.9	2.7	1.8	7.9		14.7			98.9	[94]
*L. m. rhombeata*	10.8	26.5	29.5	0.5	1.4	2.7		28			99.4	[96]
*L. stenophrys*	14.1	21.2	30.6	2.7		3.6		27.1			99.3	[94]
*Porthidium lansbergii*	16.2	4.5	35.5	3.6	1.4	6.7	12.9	12.4			93.2	[97]
*P. nasutum*	11.6	9.6	52.1	3	1.3	10.4	9.9	1.9			99.8	[81]
*P. ophryomegus*	13.5	7.3	45	3.3	0.6	8	16.7	4.2			98.6	[97]
*Rhinocerophis alternatus*	2	5.8	52.2	14.9	2.5	14.8					92.2	[70]
*R. cotiara*	0.6	13	51	19.6	2.9	4.7					91.8	[70]
*R. fonescai*	30.1	4.1	42.5	1.9	2.4	9.8	4.4				95.2	[98]

Abbreviations: **PLA_2_**, phospholipase A_2_; **SVSP**, snake venom serine protease; **SVMP**, snake venom metalloprotease; **LAAO**, l-amino acid oxidase; **CRiSP**, Cysteine-Rich Secretory Protein; **CTL/SNACLEC**, C-type lectins and C-type lectin like; **DIS**, disintegrin; **NP**, natriuretic peptides, including vasoactive peptides, bradykinin potentiating and inhibitory peptides; **DEF**, defensin (crotamine); **MPi**, snake venom metalloprotease inhibitor; **%WV**, percentage of whole venom.

## Supplementary Materials

The following are available online at www.mdpi.com/2072-6651/9/9/290/s1, Table S1: Elapid species with unusual venom composition, Table S2: The unusual venom composition of *Tropidolaemus wagleri* (Temple Pit Viper), Table S3: The remaining 36 protein families in viper and elapid venoms have been classed as rare protein families, Most have only been recorded in one or two species of snakes, and always made up less than 10% of the whole venom. Table S4: The five non-front fanged snakes included in the study with the proportion of the ten major protein families in each venom (expressed as % of total venom), which make up the majority of their venom proteome.
