# Supplementary material for: A Review and Database of Snake Venom Proteomes
Source: Toxins (Basel). 2017 Sep 18;9(9):290. doi: 10.3390/toxins9090290 (PMC5618223; doi:10.3390/toxins9090290)
Supplement: Supplementary file 1 [file toxins-09-00290-s001.pdf]

Supplementary Materials: A Review and Database of Snake Venom Proteomes

Theo Tasoulis and Geoffrey K. Isbister *

Table S1. Elapid species with unusual venom composition.

SPECIES	PLA_2	β-B	SVMP	LAAO	3FT	OHN	CRiSP	MT	CYS	PDE	% WV	REFERENCE
Bungarus multicinctus China	8.1	58.3		0.2	32.6						99.2	[1]
B.multicinctus Vietnam	15.3	40.8	0.8	2.1	27.5		2.4				88.9	[2]
Calliophis bivirgata flaviceps	41.1		18.7			14.4		22.6	0.6	1.3	98.7	[3]

Abbreviations: **PLA_2,** phospholipase A_2, **β-B,** β-bungarotoxin (PLA2 linked with a KUN), **SVMP,** snake venom metalloprotease, **LAAO,** L-amino acid oxidase, **3FT**, three-finger toxin, **OHN,** Ohanin, **CRiSP,** Cysteine-Rich Secretary Protein, **MT**, maticotoxin, **CYS,** Cystatin **PDE,** phosphodiesterase, **% WV**, percentage of whole venom.

Table S2. The unusual venom composition of *Tropidolaemus wagleri* (Temple Pit Viper[4]).

SPECIES	PLA_2	SVSP	SVMP	LAAO	LMMNLP	PDE	CTL	WAGLERIN	CYT	% WV	REF
Tropidolaemus wagleri	7.3	5.5	1.7	1.7	34.5	1	3.5	38.2	0.9	94.3	[4]

Abbreviations: **PLA_2,** phospholipase A_2, **SVSP,** snake venom serine protease, **SVMP,** snake venom metalloprotease, **LAAO,** L-amino acid oxidase, **LMMNLP,** low molecular mass non-lethal peptide, **PDE,** phosphodiesterase, **CTL,** C-type lectin, **WAGLERIN,** waglerin, **CYT,** cytotoxin, **% WV**, percentage of whole venom.

Table S3. The remaining 36 protein families in viper and elapid venoms have been classed as rare protein families. Most have only been recorded in one or two species of snakes, and always made up less than 10% of the whole venom.

Quantitatively Minor Component Protein Families	Number of Snake Species Possessing the Toxin	Maximum Total Amount (% of WV)	Species
glutaminyl cyclase	8	2	*Cryptelytrops purpureomaculatus*
aminopeptidase	6	0.8	*Bothriechis aurifer*
endonuclease	6	0.6	*Naja mossambica*
cobra venom factor	5	1.1	*Naja naja/N. kaouthia*
transferrin	3	1.8	*Hydrophis platurus*
waprin	3	1.7	*Naja nigricollis*
endopeptidase	3	1.2	*Bungarus fasciatus*
glutothione peroxidase	3	0.2	*Micrurus clarki*
kazal-type inhibitor	2	9	*Bothriechis supraciliaris*
galactose-binding protein	2	5.5	*Bothrops jararaca*
trypsinogen	2	1.2	*Bungarus fasciatus*
albumin	2	1.1	*Hydrophis platurus*
prokineticin	2	0.4	*Dendroaspis polylepis*
selectin	1	<0.3	*Naja melanoleuca*
peroxiredoxin	1	<0.1	*Crotalus atrox*
protein c activator	1	8.87	*Agkistrodon contortrix*
cholinesterase	1	6	*Naja naja*
polyglycine peptides	1	4.1	*Bothriechis supraciliaris*
Glycine-histidine rich peptide	1	7.5	*Viridovipera stejnegeri*
flavine monoamine oxidase	1	2.5	*Naja melanoleuca*
lysosomal acid lipase A	1	2.4	*Micrurus altirostris*
fibrinogenases	1	2.28	*Agkistrodon contortrix*
haemoglobins	1	2.1	*Hydrophis platurus*
neurotrophin	1	1.6	*Calloselasma rhodostoma*
aspartic protease	1	1.12	*Vipera berus*
type-B carbyoxylesterase	1	1.1	*Naja melanoleuca*

cytotoxin	1	0.9	*Tropidolaemus wagleri*
neuronal membrane glycoprotein	1	0.8	*Micrurus mosquitensis*
insulin-like growth factor	1	0.7	*Ophiophagus hannah*
sulfhydryl oxidase	1	0.4	*Calliophis bivirgata flaviceps*
aminotransferase	1	0.2	*Daboia russelii*
complement decay-accelerating factor	1	0.2	*Aipysurus laevis*
kinesin-like protein	1	0.1	*Calloselasma rhodostoma*
ribosomal protein	1	0.1	*Micrurus alleni*
multiple inositol polyphosphate phosphatase	1	0.04	*Calliophis bivirgata flaviceps*
phosphilpase A2 inhibitor	1	0.04	*Tropidolaemus wagleri*

Table S4. The five non-front fanged snakes included in the study with the proportion of the ten major protein families in each venom (expressed as % of total venom), which make up the majority of their venom proteome.

SPECIES	PLA_2	SVSP	SVMP	MMP	3FT	CRiSP	CTL	V	EGF	DEF	% WV	REFERENCE
Boiga irregularis			24.9		67.5	3.8					96.2	[5]
Cerberus rynchops			30			22	22	26			100	[6]
Dispholidus typus	7.6	5.5	74.6		6.2	5.4	0.4				99.7	[7]
Hypsiglena sp Arizona			68.7			16.7					85.4	[5]
Thamnodynastes strigats		0.7	8.2	76.2	0.2	1.9	5.4		3.9	2.8	99.3	[8]

Abbreviations: **PLA_2**, phopholipaseA_2, **SVSP**, snake venom serine protease, **SVMP**, snake venom metalloprotease. **MMP**, matrix metalloprotease, **3FT**, three-finger toxin, **CRiSP**, cysteine-rich secretory protein, **CTL**, c-type lectin, **V**, veficolin, **EGF**, epidermal growth factor, **DEF**, defensin, **% WV**, percentage of whole venom.

References

1. Ziganshin, R.H.; Kovalchuk, S.I.; Arapidi, G.P.; Starkov, V.G.; Hoang, A.N.; Nguyen, T.T.T.; Nguyen, K.C.; Shoibonov, B.B.; Tsetlin, V.I.; Utkin, Y.N. Quantitative proteomic analysis of Vietnamese krait venoms: Neurotoxins are the major components in *Bungarus multicinctus* and phospholipases A 2 in *Bungarus fasciatus*. *Toxicon* **2015**, *107*, 197–209.
2. Shan, L.L.; Gao, J.F.; Zhang, Y.X.; Shen, S.S.; He, Y.; Wang, J.; Ma, X.M.; Ji, X. Proteomic characterization and comparison of venoms from two elapid snakes (*Bungarus multicinctus* and *Naja atra*) from China. *J. Proteom.* **2016**, *138*, 83–94.
3. Tan, C.H.; Fung, S.Y.; Yap, M.K.K.; Leong, P.K.; Liew, J.L.; Tan, N.H. Unveiling the elusive and exotic: Venomics of the Malayan blue coral snake (*Calliophis bivirgata flaviceps*). *J. Proteom.* **2016**, *132*, 1–12.
4. Tan, C.H.; Tan, K.Y.; Yap, M.K.K.; Tan, N.H. Venomics of *Tropidolaemus wagleri*, the sexually dimorphic temple pit viper: Unveiling a deeply conserved atypical toxin arsenal. *Sci. Rep.* **2017**, *7*, 43237.
5. McGivern, J.J.; Wray, K.P.; Margres, M.J.; Couch, M.E.; Mackessy, S.P.; Rokyta, D.R. RNA-seq and high-definition mass spectrometry reveal the complex and divergent venoms of two rear-fanged colubrid snakes. *BMC Genom.* **2014**, *15*, 1061.
6. OmPraba, G.; Chapeaurouge, A.; Doley, R.; Devi, K.R.; Padmanaban, P.; Venkatraman, C; Velmurugan, D.; Lin, Q.; Kini, R.M. Identification of a Novel Family of Snake Venom Proteins Veficolins from *Cerberus rynchops* Using a Venom Gland Transcriptomics and Proteomics Approach. *J. Proteome Res.* **2010**, *9*, 1882–1893.
7. Pla, D.; Sanz, L.; Whiteley, G.; Wagstaff, S.C.; Harrison, R.A.; Casewell, N.R.; Calvete, J.J. What killed Karl Patterson Schmidt? Combined venom gland transcriptomic, venomic and antivenomic analysis of the South African green tree snake (the boomslang), *Dispholidus typus*. *BBA-Gen. Subj.* **2017**, *1861*, 814–823.
8. Ching, A.T.C.; Paes Leme A.F.; Zelanis, A.; Rocha, M.M.T.; Furtado, M.d.F.D.; Silva, D.A.; Trugilho, M.R.O.; Rocha, S.L.G.d.; Perales, J.; Ho, P.L. Venomics Profiling of *Thamnodynastes strigatus* Unveils Matrix Metalloproteinases and Other Novel Proteins Recruited to the Toxin Arsenal of Rear-Fanged Snakes. *J. Proteome Res.* **2012**, *11*, 1152–1162.
